# A Rare Case of Fibrous Dysplasia Presenting With Facial Swelling and Craniofacial Deformity in a 13-Year-Old Girl

**DOI:** 10.7759/cureus.59327

**Published:** 2024-04-29

**Authors:** Rishabh Dhabalia, Shivali V Kashikar, Pratapsingh Parihar, Suhit Naseri

**Affiliations:** 1 Radiodiagnosis, Jawaharlal Nehru Medical College, Datta Meghe Institute of Higher Education and Research, Wardha, IND; 2 Radiology, Jawaharlal Nehru Medical College, Datta Meghe Institute of Higher Education and Research, Wardha, IND; 3 Pathology, Jawaharlal Nehru Medical College, Datta Meghe Institute of Higher Education and Research, Wardha, IND

**Keywords:** pediatric orthopedics, woven bone, computed tomography, facial swelling, craniofacial deformity, fibrous dysplasia

## Abstract

Fibrous dysplasia (FD) is a rare benign skeletal disorder that replaces normal bone with fibrous tissue and immature woven bone. We present a case of a 13-year-old girl with right-sided facial swelling and craniofacial deformity since birth, accompanied by nasal obstruction and difficulty in breathing and swallowing. Computed tomography (CT) imaging revealed an expansile bony lesion with a ground-glass matrix involving multiple craniofacial bones. Histopathological examination confirmed the diagnosis of FD. Management involved regular monitoring and conservative measures, with surgical intervention reserved for symptomatic progression or cosmetic concerns. This case underscores the importance of considering FD in the differential diagnosis of craniofacial asymmetry and highlights the collaborative approach to patient care. Further research is needed to optimize management strategies and outcomes for pediatric patients with FD.

## Introduction

Fibrous dysplasia (FD) is a rare benign skeletal disorder that replaces normal bone with fibrous tissue and immature woven bone. It predominantly affects children and adolescents, with a reported incidence of one in 30,000 individuals [1]. FD can present as either a monostotic form involving a single bone or a polyostotic form affecting multiple bones [2]. While monostotic FD is more common, affecting approximately 70-80% of cases, polyostotic FD tends to be more severe and can be associated with various endocrine abnormalities, including precocious puberty and hyperthyroidism [3]. FD commonly involves the craniofacial bones, including the maxilla, mandible, frontal, and sphenoid bones, leading to characteristic clinical features such as facial asymmetry, swelling, and deformity [4]. The clinical presentation of FD can vary widely depending on the site and extent of bone involvement, ranging from asymptomatic incidental findings to significant functional impairments. Nasal obstruction, difficulty breathing, and swallowing difficulties can occur when the lesion affects the paranasal sinuses or adjacent structures [5].

Imaging modalities, including computed tomography (CT) and magnetic resonance imaging (MRI), play a crucial role in diagnosing and evaluating FD. CT imaging typically reveals expansile bony lesions with a ground-glass matrix appearance, while MRI can provide additional information about the lesion's soft tissue involvement and extension [3]. Histopathological examination remains the gold standard for confirming the diagnosis of FD, demonstrating the presence of fibrous tissue intermingled with immature woven bone without conspicuous osteoblastic rimming [6]. Management of FD depends on various factors, including the extent of bone involvement, symptoms, and complications. Conservative management with regular monitoring may be sufficient for asymptomatic or minimally symptomatic cases, while surgical intervention may be required for symptomatic or cosmetically significant lesions [7]. In this report, we present a case of fibrous dysplasia in a 13-year-old girl, highlighting the characteristic clinical and radiological features of the condition and emphasizing the importance of accurate diagnosis and appropriate management strategies.

## Case presentation

**Figure 1:**
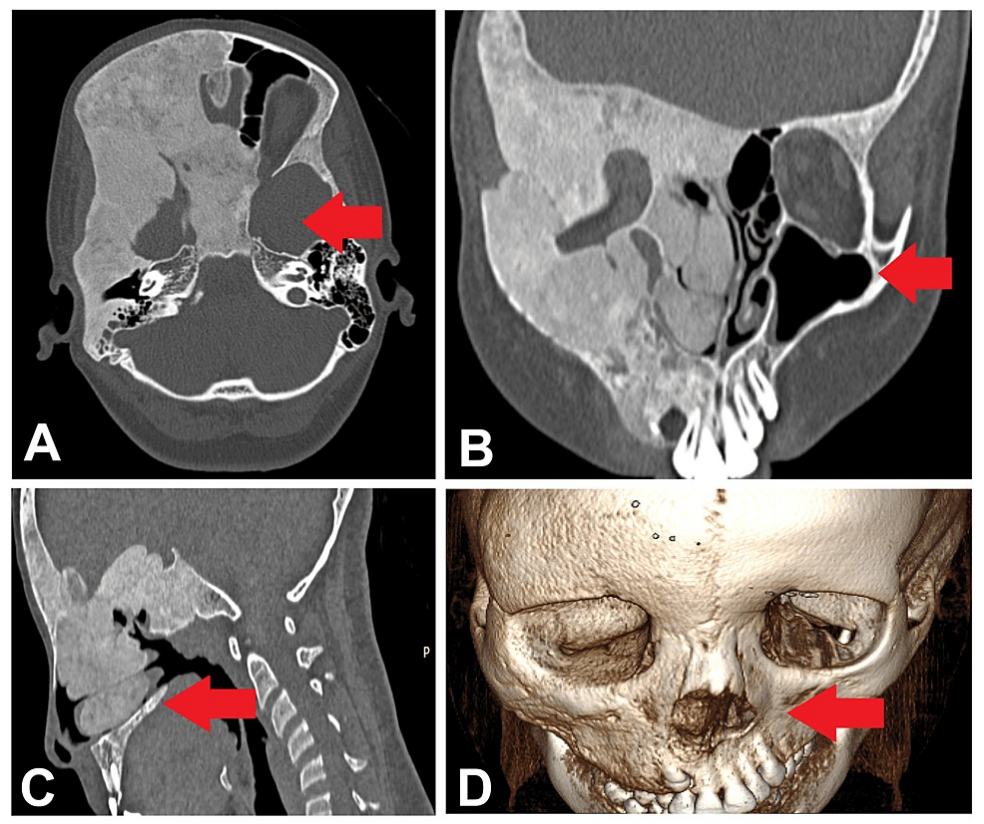
Images of paranasal sinuses CT images of paranasal sinuses, axial coronal (A), sagittal (B), bone window (C), and third volume rendered image (D) showing expansile bony lesion with ground glass matrix involving right frontal, parietal and temporal bones, greater wing of sphenoid, right medial and lateral pterygoid plates and diaphragma sellae. It is causing narrowing of the right frontal, ethmoid, maxillary, and sphenoid sinuses and right nasal cavity.

A 13-year-old girl presented to our clinic with a complaint of right-sided facial swelling and asymmetry, which was noticed since birth. Additionally, she reported experiencing nasal obstruction and difficulty breathing and swallowing. On physical examination, there was evident asymmetry of the right side of her face, characterized by prominence of the right cheek and jaw. Notably, there was no associated pain or tenderness upon palpation. Nasal endoscopy revealed deviation of the nasal septum towards the left side, accompanied by narrowing of the right nasal cavity. Further investigation through computed tomography (CT) imaging of the paranasal sinuses was performed to evaluate the extent and nature of the facial asymmetry. The CT scans revealed an expansive bony lesion involving several craniofacial bones, including the right frontal, parietal, and temporal bones, as well as the greater wing of the sphenoid bone, right medial and lateral pterygoid plates, and diaphragma sellae. The lesion exhibited a distinctive ground-glass matrix appearance, significantly narrowing the right frontal, ethmoid, maxillary, and sphenoid sinuses, along with the right nasal cavity (Figure 1).

**Figure 2:**
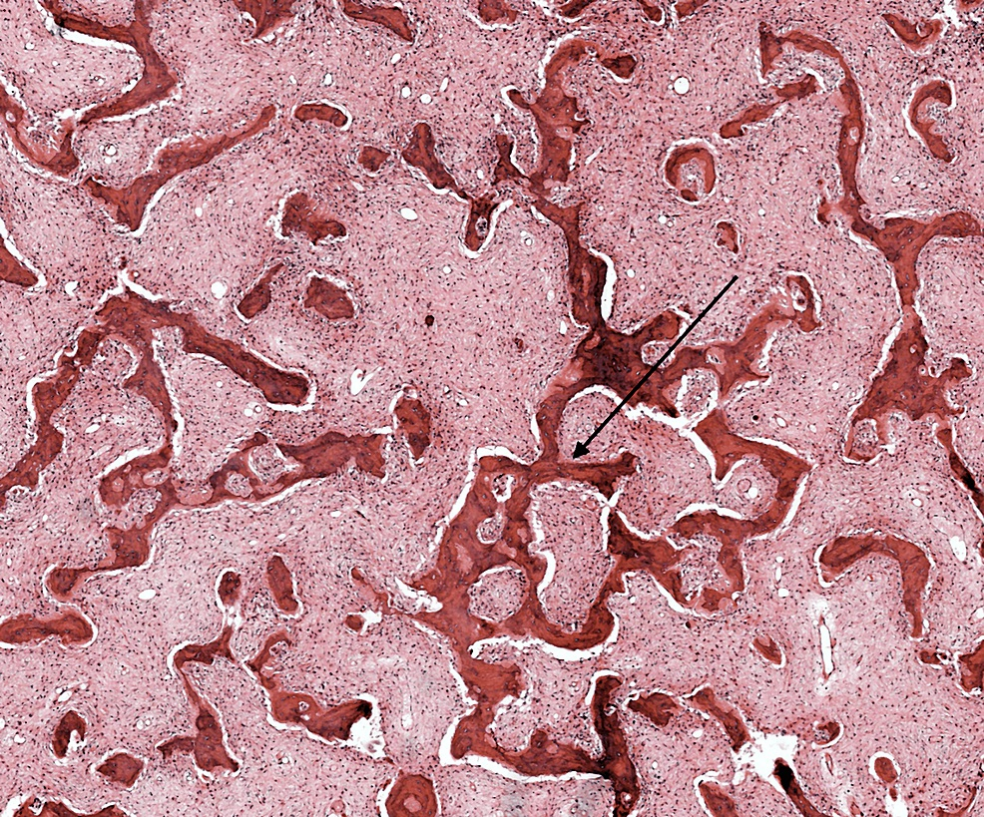
Histopathological examination The image shows woven bone within the lesion that appeared as irregularly shaped "C" or "S" trabeculae with anastomosing patterns, lacking conspicuous osteoblastic rimming.

The woven bone within the lesion appeared as irregularly shaped "C" or "S" trabeculae with anastomosing patterns, lacking conspicuous osteoblastic rimming (Figure 2). Based on the clinical presentation and imaging findings, a provisional diagnosis of fibrous dysplasia was considered. A biopsy specimen was obtained from the lesion for histopathological examination to confirm the diagnosis. The histopathological analysis revealed the presence of fibrous tissue intermingled with immature woven bone, consistent with the characteristic features of fibrous dysplasia. In terms of management, a conservative approach was adopted, with regular followup visits scheduled to monitor the lesion's progression and manage symptomatic complaints such as nasal obstruction. Surgical intervention was not deemed necessary at the time of presentation due to the asymptomatic nature of the lesion and the absence of functional impairments. The patient and her family were educated about the condition and reassured regarding the benign nature of fibrous dysplasia.

## Discussion

FD is a rare benign skeletal disorder that replaces normal bone with fibrous tissue and immature woven bone. It can present as a monostotic or polyostotic form, affecting a single bone or multiple bones, respectively, with a predilection for the craniofacial region, long bones, ribs, and pelvis [3]. The case presented here highlights the clinical and radiological features of FD in a 13-year-old girl with right-sided facial swelling and craniofacial deformity since birth. The characteristic clinical manifestations of FD in the craniofacial region include facial asymmetry, swelling, and functional impairments such as nasal obstruction, difficulty breathing, and swallowing [8]. Consistent with previous literature, our patient exhibited prominent asymmetry on the right side of her face and reported nasal obstruction and difficulty breathing and swallowing [9].

Imaging modalities, particularly computed tomography (CT) and magnetic resonance imaging (MRI), play a crucial role in diagnosing and evaluating FD. CT imaging typically reveals expansile bony lesions with a ground-glass matrix appearance involving multiple craniofacial bones, as seen in our case [10]. The presence of irregularly shaped trabeculae with anastomosing patterns, characteristic of woven bone, is also a hallmark of FD on imaging [1]. Furthermore, MRI can provide additional information regarding soft tissue involvement and the extent of the lesion. Histopathological examination remains the gold standard for confirming the diagnosis of FD. The presence of fibrous tissue intermingled with immature woven bone, as observed in our patient's biopsy specimen, is consistent with the histological features of FD [11]. Management of FD depends on various factors, including the extent of the lesion, symptoms, and functional impairments. Conservative management with regular monitoring may be sufficient in asymptomatic cases or those with mild symptoms [12]. Surgical intervention may be considered for symptomatic or cosmetically significant lesions and for preventing complications such as fractures or compression of adjacent structures [13].

## Conclusions

In conclusion, this case report sheds light on the clinical presentation, radiological features, and management considerations of FD in the craniofacial region. Through a multidisciplinary approach involving clinical evaluation, imaging modalities, and histopathological analysis, a definitive diagnosis of FD was established in a 13-year-old girl presenting with right-sided facial swelling and craniofacial deformity since birth. The characteristic findings of an expansile bony lesion with a ground-glass matrix appearance on CT imaging, accompanied by irregularly shaped trabeculae of woven bone, were consistent with the diagnosis. Management strategies focused on regular monitoring and conservative measures, given the asymptomatic nature of the lesion and the absence of functional impairments. Surgical intervention was deferred unless warranted by symptomatic progression or cosmetic concerns. This case underscores the importance of considering FD in the differential diagnosis of craniofacial asymmetry and highlights the collaborative efforts of healthcare professionals in optimizing patient care and outcomes. Further research and long-term follow-up studies are warranted to enhance our understanding of the natural history and optimal management strategies for FD in pediatric patients.
